# 18F-FDG PET-Derived Textural Indices Reflect Tissue-Specific Uptake Pattern in Non-Small Cell Lung Cancer

**DOI:** 10.1371/journal.pone.0145063

**Published:** 2015-12-15

**Authors:** Fanny Orlhac, Michaël Soussan, Kader Chouahnia, Emmanuel Martinod, Irène Buvat

**Affiliations:** 1 Imagerie Moléculaire In Vivo, IMIV, CEA, Inserm, CNRS, Univ. Paris-Sud, Université Paris Saclay, CEA-SHFJ, Orsay, France; 2 Department of Nuclear Medicine, AP-HP, Avicenne Hospital, Bobigny, France; 3 Department of Oncology, AP-HP, Avicenne Hospital, Bobigny, France; 4 Department of Thoracic Surgery, AP-HP, Avicenne Hospital, Bobigny, France; Memorial Sloan-Kettering Cancer Center, UNITED STATES

## Abstract

**Purpose:**

Texture indices (TI) calculated from 18F-FDG PET tumor images show promise for predicting response to therapy and survival. Their calculation involves a resampling of standardized uptake values (SUV) within the tumor. This resampling can be performed differently and significantly impacts the TI values. Our aim was to investigate how the resampling approach affects the ability of TI to reflect tissue-specific pattern of metabolic activity.

**Methods:**

18F-FDG PET were acquired for 48 naïve-treatment patients with non-small cell lung cancer and for a uniform phantom. We studied 7 TI, SUVmax and metabolic volume (MV) in the phantom, tumors and healthy tissue using the usual relative resampling (RR) method and an absolute resampling (AR) method. The differences in TI values between tissue types and cancer subtypes were investigated using Wilcoxon’s tests.

**Results:**

Most RR-based TI were highly correlated with MV for tumors less than 60 mL (Spearman correlation coefficient r between 0.74 and 1), while this correlation was reduced for AR-based TI (r between 0.06 and 0.27 except for RLNU where r = 0.91). Most AR-based TI were significantly different between tumor and healthy tissues (pvalues <0.01 for all 7 TI) and between cancer subtypes (pvalues<0.05 for 6 TI). Healthy tissue and adenocarcinomas exhibited more homogeneous texture than tumor tissue and squamous cell carcinomas respectively.

**Conclusion:**

TI computed using an AR method vary as a function of the tissue type and cancer subtype more than the TI involving the usual RR method. AR-based TI might be useful for tumor characterization.

## Introduction

Characterizing tumor heterogeneity is a challenge in oncology. As explained by Marusyk and Polyak [1], the coexistence of different clones in a tumor affects the evolution of cancer and should be accounted for in patient management. Measuring the extent of clonal heterogeneity could therefore be extremely useful to select the most promising therapeutic approach. Several groups have characterized tumor heterogeneity using textural indices in 18F-FDG PET [2–12] but contradictory results have been reported. For instance, in head and neck cancer, Cheng et al [8] showed that a tumor uniformity index (also called energy in other papers) calculated in pretreatment 18F-FDG PET images was a prognostic factor in advanced T-stage, while El Naqa [3] could not predict patient response using this index. Moreover, most analyses used a univariate approach and did not actually assess the incremental value of textural metrics over conventional indices such as tumor metabolic volume or FDG uptake. Also, it has been pointed out that textural metrics should be used with caution, as some metrics are substantially variable with respect to the acquisition mode and reconstruction parameters [13], are strongly correlated with the tumor metabolic volume [14,15] and/or are redundant [14]. Misconceptions regarding the meaning of textural indices derived from PET images have been recently underlined, especially given the instrumental limitations encountered in PET [16,17]. In a recent study using 18F-FDG PET data, Brooks and Grigsby [15] demonstrated that below a tumor volume of 45 cm^3^ (corresponding to 700 voxels in their work), the entropy metrics mostly reflected the tumor volume and not the heterogeneity of tracer uptake in the tumor. This result therefore questions the use of textural metrics for a broad range of tumors and casts doubt upon previous conclusions obtained in tumors for which the volume was not reported. Last, many studies that demonstrated the ability of TI to distinguish between tumor groups did actually not properly control the false positive rate caused by the large number of hypotheses tested simultaneously [18]. Moreover, the fact that FDG-PET images can depict tissue-specific textural pattern, meaning that different tissues might be characterized by different TI values, has received little attention in the literature. Similarly, the meaning and actual variation of TI as a function of tissue type has not been thoroughly investigated in FDG-PET.

TI calculation requires an initial resampling of the tumor voxel intensities that sets voxels with similar uptake to the same value hence reduces the impact of noise. The usual resampling method consists in rescaling the tumor voxel values between 0 and 1 using a fixed number of bins (typically 64), so that the maximum voxel value in the tumor is set to 1 while the minimum is set to 0. Alternative resampling schemes have been proposed [19,20]. By definition, this resampling step affects the value of TI. Yet, the impact of this resampling on the ability of TI to reflect useful information regarding the uptake spatial distribution has been overlooked. The goal of our study was therefore to investigate the impact of this resampling step on TI values and on the ability of TI to reflect tissue-specific pattern of metabolic activity. For that purpose, we first used a uniform FDG-filled phantom to characterize the behavior of TI as a function of the region volume when no biological texture is present for two resampling approaches. Second, we analyzed images from patients with non-small cell lung cancer (NSCLC) to confirm the findings obtained using the phantom and to determine whether the regional FDG uptake pattern measured using different TI could distinguish between tissue types and whether this distinction depended on the resampling scheme.

## Materials and Methods

### Phantom and patients

A 20 cm high cylinder (16 cm in diameter) was filled with water including 103.3 MBq 18F-FDG. For image acquisitions, the cylinder axis was parallel to the axial direction of the PET/CT scanner.

**Fig 1:**
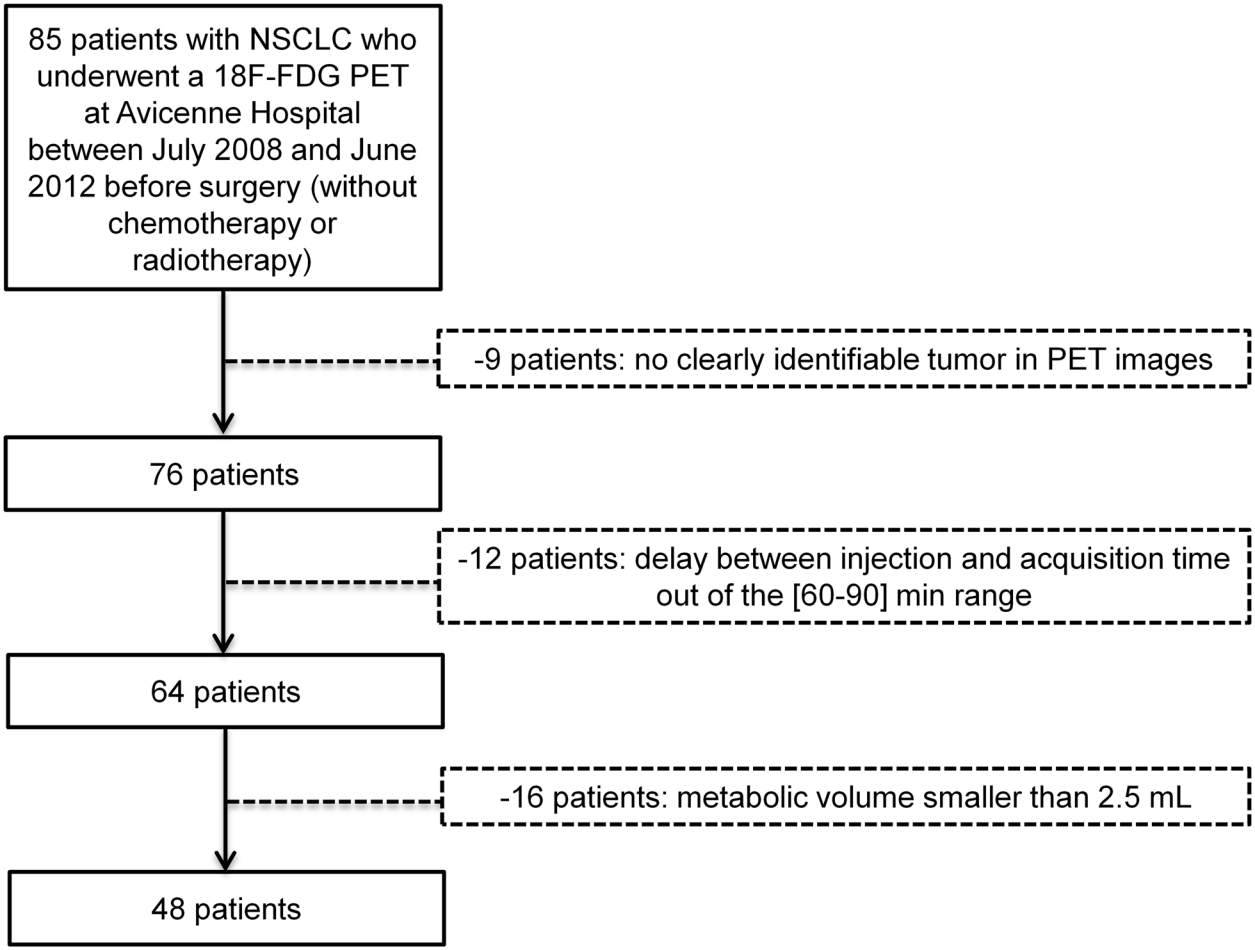
Selection criteria to identify the 48 patients with NSCLC.

Patient data were retrospectively retrieved after approval by the local institution review board of Avicenne Hospital (Ile-de-France X), and the requirement to obtain informed consent was waived. Patient information was anonymized and de-identified prior to analysis. Eighty-five consecutive patients with NSCLC underwent a 18F-FDG PET/CT between July 2008 and June 2012 before surgery. Among them, only 48 patients were included in the study, whose characteristics are summarized in Table 1. As detailed in Fig 1, the exclusion criteria were: 1) no clearly identifiable tumor in PET images (n = 9 patients), 2) delay between injection time and acquisition time departing from the [60–90] min range (n = 12 patients) and 3) metabolic volume after segmentation lower than 2.5 mL (n = 16 patients).

**Table 1:** Patient characteristics.

Characteristic		Patients
**Sex**	Male	34
	Female	14
**Age (y)**	Mean	64
	Range	[48–85]
**Histology**	Adenocarcinoma	28
	Squamous cell carcinoma	13
	Other	7
**T stage**	T1	12
	T2	28
	T3	6
	T4	2
**N stage**	N0	33
	N1	7
	N2	8

All 48 patients underwent an 18F-FDG PET/CT scan before the start of therapy. The PET/CT scans were performed at 75±9 min (range: 60–90 min) after injection of 18F-FDG (3–3.5 MBq/kg). For all patients, the serum glucose level was less than 1.4 g/L at the time of injection. All patients underwent surgery, and histological analysis was performed on the tumor specimen to determine the subtype of cancer (Table 1). Among 48 patients, 28 patients had an adenocarcinoma, 13 had a squamous cell carcinoma while 7 had other NSCLC types. The majority of patients had a T2 primary lesion, 15 patients had lymph node metastases. No patient had distant metastasis or a previously identified liver pathology.

### PET/CT acquisitions

All 18F-FDG PET/CT images were obtained using a Gemini TF PET/CT scanner (Philips). The PET scans were performed using 1.45 min per bed position acquisitions (scanner axial field-of-view of 18 cm) and 8 to 10 bed positions for patients and 3 min for one bed position for the phantom. PET images were reconstructed using a list-mode iterative algorithm (BLOB-OS-TF, 2 iterations, 33 subsets) and the voxel size was 4 mm x 4 mm x 4 mm. A single scatter-simulation model was used for scatter correction. For attenuation correction, a low-dose CT scan was acquired (no post-reconstruction smoothing). CT images were obtained without contrast agent, with an X-ray tube voltage peak of 120 kV and 140 kV, 100 mAs and 32 mAs respectively for patients and phantom, a 0.69 pitch, a slice thickness of 2 mm and increment of 1 mm. The voxel size for CT was 0.68 mm x 0.68 mm x 0.68 mm. PET images were converted in SUV units normalized by the patient body weight.

### Volume of Interest delineation

**Fig 2:**
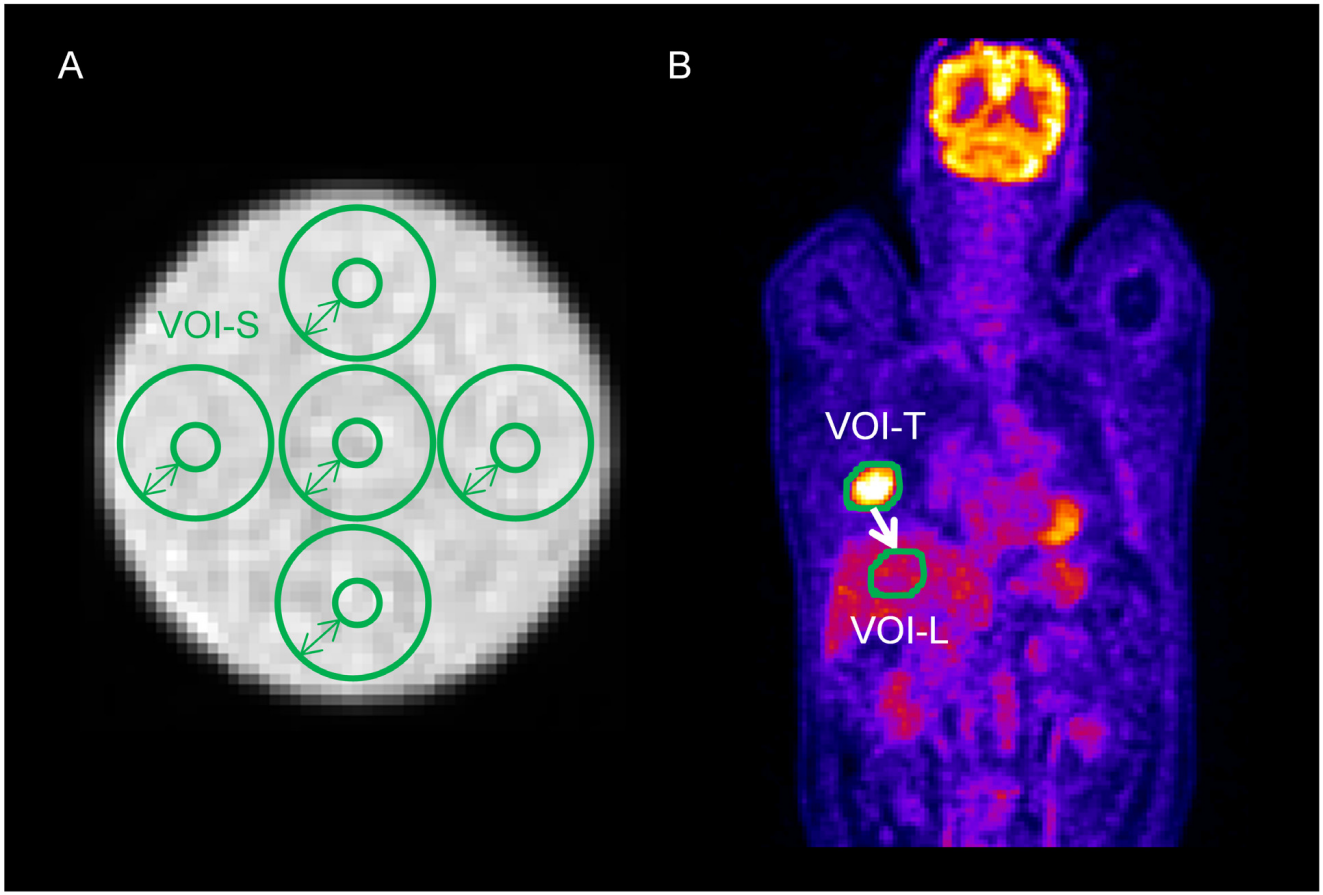
Examples of segmentation. Delineation of spheres in the phantom data (A). Segmentation of tumors in a NSCLC patient (B) and relocation of the tumor volume in the liver of the patient.

Five spherical volumes of interest (VOI-S) centered on 5 different positions were automatically drawn on phantom PET images (Fig 2A). The sphere diameter was varied from 3 to 17 voxels (only odd values were used to center the sphere on a voxel) so as to study the impact of the region volume on TI values.

For each patient, the lung tumor was delineated using an adaptive threshold [21] as described previously [14], resulting in a VOI-T volume. This VOI (VOI-T) was then moved well inside the liver (VOI-L) of the patient (Fig 2B), that was supposed to be a healthy tissue region of reference. This liver region of reference was preferred to a contralateral healthy lung region given the very low FDG uptake in normal lung tissue.

### Resampling methods

As mentioned before, an initial step to compute TI is to resample voxel intensities using a fixed number of discrete values. This step sets voxels with close intensities to the same value, thus reducing the impact of noise and the size of the matrices needed for subsequent TI calculation. Most previous publications resampled the tumor values between the minimum (SUVmin) and the maximum (SUVmax) intensity in the tumor VOI, using:

$$R1\left(x\right)=round\left(D\times \frac{I\left(x\right)-SUVmin}{SUVmax-SUVmin}\right)$$ (1)

where R1(x) is the resampled value in voxel x, I(x) is the value in voxel x before resampling and D the number of discrete values, often taken equal to 64. This resampling is called Relative Resampling (RR) thereafter. To overcome the limitations of this RR approach (see Results section), we tested an original Absolute Resampling (AR) method in which the bounds used for resampling were set to 0 and 20 SUV units corresponding to the typical range of tumor SUVs in our patients, so that:

$$R2\left(x\right)=round\left(D\times \frac{I\left(x\right)}{20}\right)$$ (2)

For each VOI, we resampled the voxel intensities using RR and AR, choosing D equal to 64 discrete values [14].

To check the robustness of the results as a function of the high bound in the AR method, we also computed the TI using the AR method with the high bound set to 15 or 25 SUV units instead of 20 SUV units. These three approaches will be denoted AR15, AR20 and AR25 in the following.

### Texture analysis

For each VOI, metabolic volume (MV) and maximum SUV (SUVmax) were computed. After RR or AR resampling, three texture matrices were calculated as explained in [14]: the co-occurrence matrix (CM), the gray-level run length matrix (GRLM) and the gray-level zone length matrix (GZLM). The co-occurrence matrix was computed in 13 directions using a distance of 1 voxel, and each TI calculated from this matrix corresponds to the average value in the 13 directions. Similarly, the GRLM was calculated for 13 directions while the GZLM was computed directly in 3 dimensions.

Seven TI were derived per VOI using the home-made LIFEx (*Local Image Features Extraction*) software, by selecting one index robust to segmentation in each group of highly correlated indices previously identified in [14]: homogeneity and entropy from CM, Short-Run Emphasis (SRE), Long-Run Emphasis (LRE) and Run Length Non-Uniformity (RLNU) based on GRLM, and Low Gray-Level Zone Emphasis (LGZE) and High Gray-level Zone Emphasis (HGZE) from GZLM.

### Statistical analysis

To study the impact of the resampling methods on the relationship between TI and region volume or metabolic activity, we plotted all TI (mean and standard deviation over the 5 sphere positions in the phantom) as a function of the number of voxels in VOI-S, VOI-T and VOI-L, and as a function of SUVmax. These plots were characterized by the Spearman correlation coefficient (r) between the TI and the region volume or between the TI and the region SUVmax.

To determine whether the TI could distinguish between different uptake spatial distributions in different tissue types, we used Wilcoxon’s tests. Tests were performed for each TI and for the two resampling methods to distinguish between 1) lung tumors and healthy tissue and 2) adenocarcinoma and squamous cell carcinoma.

## Results

### Relationship between RR-based TI and volume or SUVmax

The mean tumor volume segmented by the adaptive thresholding method was 19.5±26.1 ml (range:[2.8–119.0 ml]).

**Fig 3:**
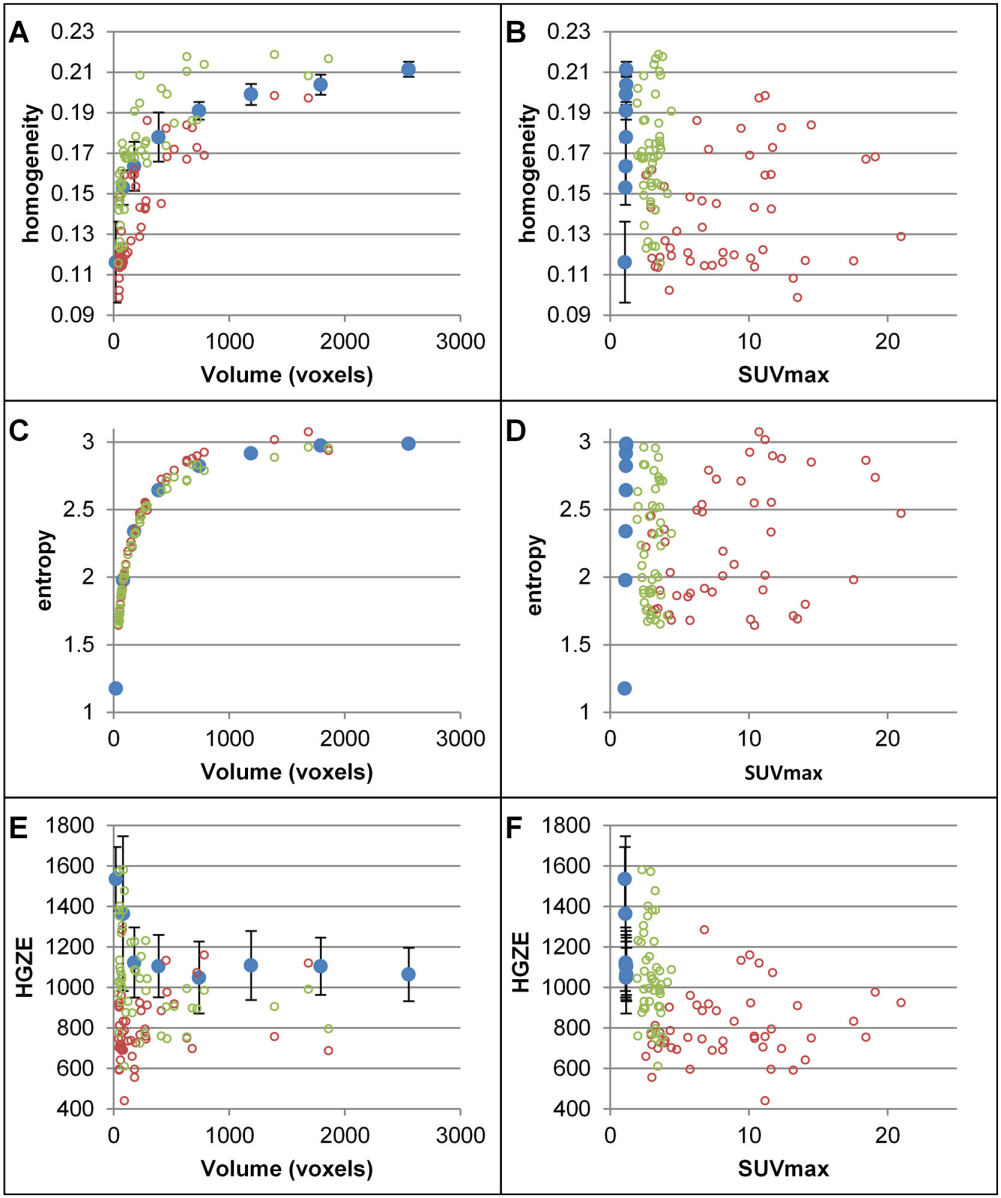
RR-based TI as function of MV and SUVmax. Plots of homogeneity (A-B), entropy (C-D) and HGZE (E-F) as a function of the number of voxels (A, C, E) or as a function of SUVmax (B, D, F) for the phantom (blue), lung tumors (red) and healthy tissue (green) with the RR method for TI calculation.

The plots of RR-based TI as a function of volume (Fig 3 and S1 Fig) show that most indices increase or decrease at the beginning of the curve and then reach a plateau. The same trend is observed for the phantom data, the lung tumors and the healthy tissue. The trends seen in Fig 3A and 3C and S1A, S1C and S1G Fig show that these TI are strongly correlated with MV for small VOIs (|r| between 0.74 and 0.99 for VOI-T and VOI-L volumes). HGZE exhibits another trend with large fluctuations for small VOIs (Fig 3E). RLNU increases linearly with the number of voxels and never reaches a plateau (S1E Fig) (r = 1). The plots of TI as a function of SUVmax (Fig 3B, 3D and 3F and S1B, S1D, S1F and S1H Fig) demonstrate that RR-based TI are independent of the uptake in the VOI (|r| between 0.01 and 0.43).

### Relationship between AR-based TI and volume or SUVmax

**Fig 4:**
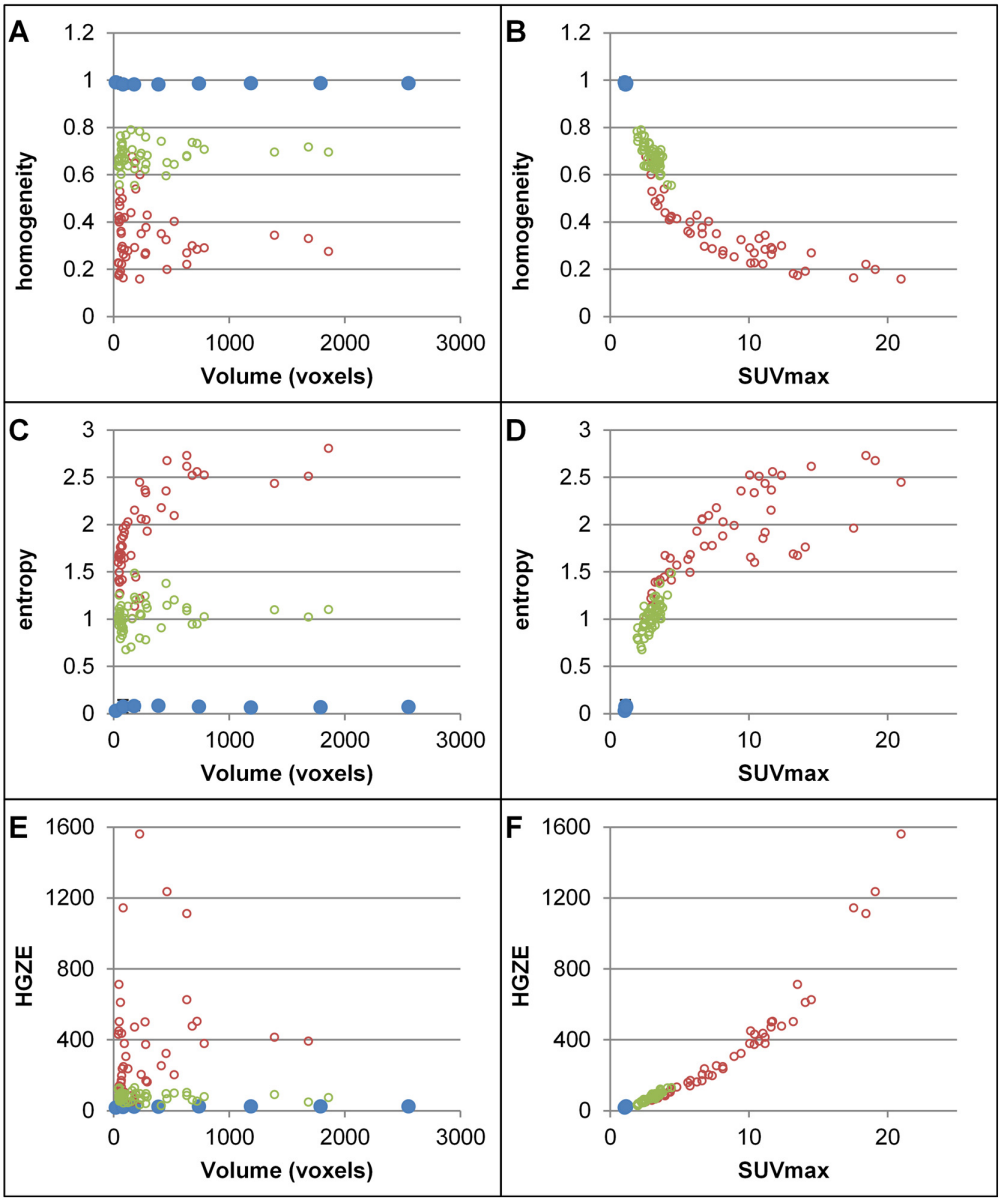
AR-based TI as a function of MV and SUVmax. Plots of homogeneity (A-B), entropy (C-D) and HGZE (E-F) as a function of the number of voxels (A, C, E) or as a function of SUVmax (B, D, F) for the phantom (blue), lung tumors (red) and healthy tissue (green) with the AR20 method for TI calculation.

The correlation coefficients between AR-based TI and volume (Fig 4A, 4C and 4E and S2A, S2C and S2G Fig) were much lower than with RR-based TI, with |r| between 0.06 and 0.27, except for RLNU (S2E Fig, r = 0.91). Yet, AR-based TI were correlated with SUVmax with |r| between 0.47 and 0.98, except for phantom data for which SUVmax was almost constant.

### TI differences in tumors and healthy tissue

**Fig 5:**
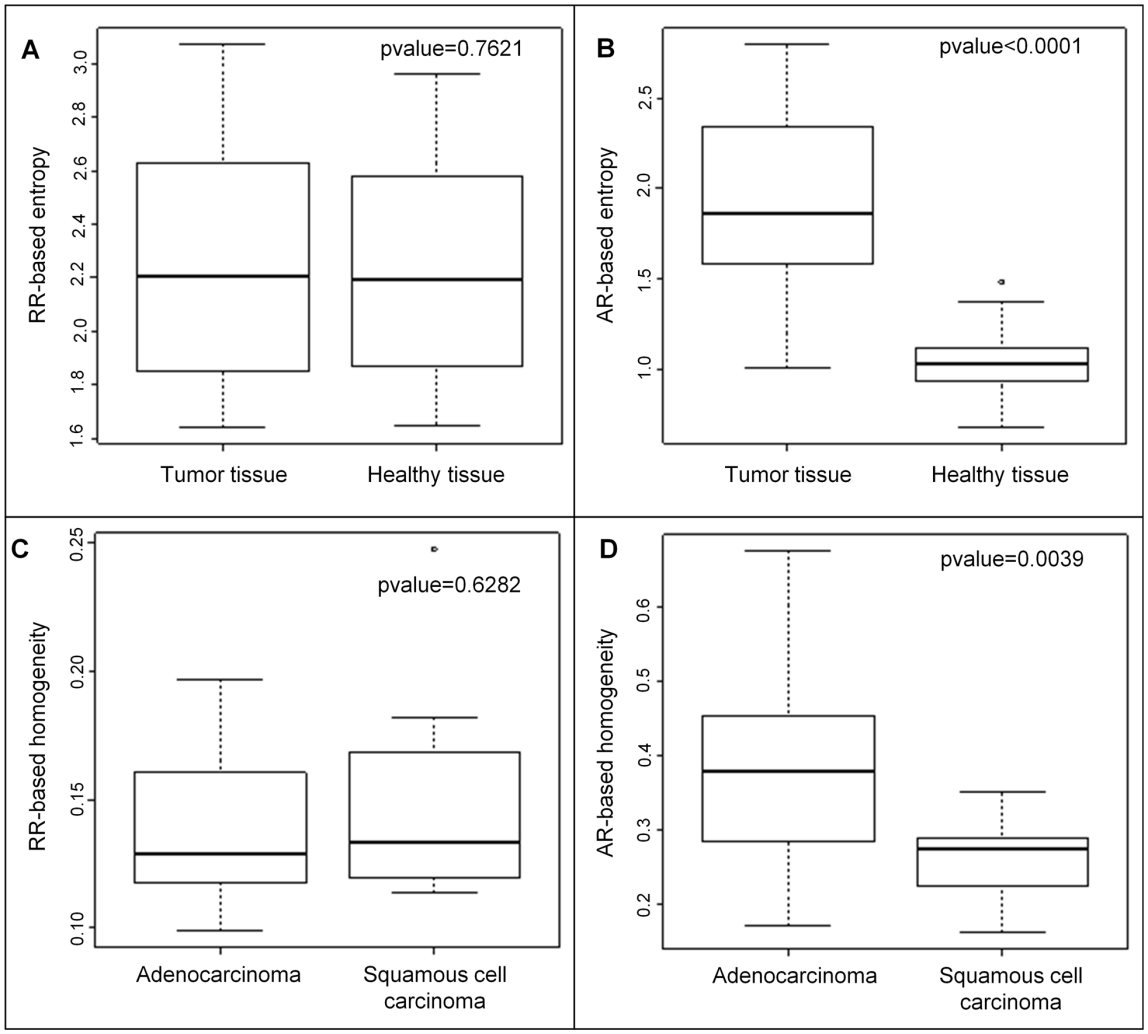
Comparison of RR and AR-based TI. Boxplots of RR- based entropy (A) and AR-based entropy (B) for tumor and healthy tissue and RR-based homogeneity (C) and AR-based homogeneity (D) for adenocarcinoma and squamous cell carcinoma and the pvalues of associated Wilcoxon’s tests.

We compared the values of the RR-based TI and AR-based TI in tumor and healthy tissue. The pvalues of Wilcoxon’s test are reported in Table 2 and mean value, first and third quartiles are given in S1 Table for the two tissue types. Homogeneity, SRE, LRE, HGZE and LGZE were significantly different (p<0.05) between tumor and healthy tissue regions, but none of the pvalues calculated for an RR-based TI was lower than that obtained for SUVmax, suggesting that none of the RR-based TI did better than SUV at separating tumor from healthy tissue regions. With the AR-based TI, the pvalues were always lower than those obtained for RR-based TI, suggesting a better identification of tissue types. Homogeneity, entropy, SRE and LRE yielded a pvalue lower than that of SUVmax. For instance, as shown in Fig 5A and 5B, RR-based entropy did not distinguish between tumor and healthy tissue (pvalue = 0.7621) whereas the same index computed with AR method could differentiate between these two tissue types (pvalue<0.0001). Results were almost unchanged between AR15, AR20 and AR25 (Table 2).

**Table 2:** Pvalues of Wilcoxon’s test for RR-based and AR-based texture indices between tumor tissue and healthy tissue.

Index	Relative resampling (RR)	Absolute resampling (AR20) High bound: SUV = 20	Absolute resampling (AR15) High bound: SUV = 15	Absolute resampling (AR25) High bound: SUV = 25
Homogeneity	**7.76E-06**	**< 2.2E-16**	**< 2.2E-16**	**< 2.2E-16**
Entropy	0.7621	**< 2.2E-16**	**< 2.2E-16**	**< 2.2E-16**
SRE	**0.0488**	**< 2.2E-16**	**< 2.2E-16**	**< 2.2E-16**
LRE	**0.0424**	**< 2.2E-16**	**< 2.2E-16**	**< 2.2E-16**
RLNU	0.8869	**0.0011**	**0.0116**	**0.0002**
LGZE	**6.67E-06**	**1.94E-11**	**2.35E-11**	**1.53E-09**
HGZE	**8.67E-09**	**2.06E-12**	**1.92E-12**	**2.49E-10**
SUVmax	**3.56E-16**	**3.56E-16**	**3.56E-16**	**3.56E-16**

Pvalues in bold are lower than 5%.

Using the AR method (Table 3), tumor tissue exhibited higher HGZE than healthy tissue, while tumors had lower homogeneity and LGZE.

**Table 3:** Summary of AR-based TI differences between tumor and healthy tissue and between adenocarcinoma and squamous cell carcinoma.

Index	Tumor tissue	Healthy tissue	Adenocarcinoma	Squamous cell carcinoma
Homogeneity	-	+	+	-
Entropy	+	-	-	+
SRE	+	-	-	+
LRE	-	+	+	-
RLNU	+	-	-	+
LGZE	-	+	+	-
HGZE	+	-	-	+
SUVmax	+	-	-	+
MV	N/A	N/A	-	+

N/A: not applicable

+: high in this tissue

-: low in this tissue

For instance (first row), homogeneity is higher in healthy tissue than in tumor tissue, and is also higher in adenocarcinoma than in squamous cell carcinoma.

### TI differences between adenocarcinomas and squamous cell carcinomas

The results of Wilcoxon’s test performed to determine whether TI are significantly different in adenocarcinomas (n = 28) and squamous cell carcinomas (n = 13) are summarized in Table 4. All RR-based TI yielded pvalues higher than 0.07 while the AR-based TI always led to pvalues lower than or equal to 5%. For example, homogeneity computed with RR method was not significantly different between the two subtypes of cancer while it was with AR method (Fig 5C and 5D). The pvalues observed for homogeneity (pvalue = 0.0039), entropy (pvalue = 0.0039), LGZE (pvalue = 0.0083) and HGZE (pvalue = 0.0076) are lower than those obtained for SUVmax (pvalue = 0.0108) and MV (pvalue = 0.1303) between the two subtypes of cancer with the AR20 method. The adenocarcinomas show a higher homogeneity and lower entropy than the squamous cell carcinomas (Table 3 and S2 Table). These results remained very similar for the AR15 and AR25 resampling schemes.

**Table 4:** Pvalues of Wilcoxon’s test for RR-based and AR-based texture indices between adenocarcinoma and squamous cell carcinoma.

Index	Relative resampling (RR)	Absolute resampling (AR20) High bound: SUV = 20	Absolute resampling (AR15) High bound: SUV = 15	Absolute resampling (AR25) High bound: SUV = 25
Homogeneity	0.6282	**0.0039**	**0.0245**	**0.0035**
Entropy	0.1143	**0.0039**	**0.0179**	**0.0029**
SRE	0.1765	**0.0179**	0.0619	**0.0227**
LRE	0.1953	0.0506	0.1210	**0.0357**
RLNU	0.1210	**0.0473**	0.0849	**0.0383**
LGZE	0.3823	**0.0083**	**0.0083**	**0.0069**
HGZE	0.0799	**0.0076**	**0.0076**	**0.0076**
SUVmax	**0.0108**	**0.0108**	**0.0108**	**0.0108**
MV	0.1303	0.1303	0.1303	0.1303

Pvalues in bold are lower than 5%.

## Discussion

In this study, we showed that TI computed using an AR method were less correlated to MV than those calculated with a RR method and demonstrated that AR-based TI were significantly different in healthy and tumor tissue or in two subtypes of lung tumors. These differences were higher than those observed with SUVmax, MV and RR-based TI. We found that lung tumor tissue exhibited more heterogeneous texture than healthy tissue, corresponding to lower homogeneity and higher entropy (Table 3). Similarly, we observed that squamous cell carcinomas yielded a more heterogeneous tumor metabolism than adenocarcinomas.

The first objective of our study was to determine the impact of the resampling method on the resulting TI and on their correlation with the region volume and SUVs. Unlike Brooks and Grigsby [15], we used PET images of a phantom uniformly filled with FDG for our experiments. For that phantom, the texture of the FDG distribution was only that of the PET signal which is spatially correlated in the reconstructed images [22], as there was no “physiological” texture in the phantom. The behavior of TI as a function of the volume of the region used to calculate it could thus be investigated without any confounding factor potentially introduced by an underlying physiological signal. When using the usual RR resampling, we found that change in entropy (Fig 3C) with the VOI volume was very similar to that previously reported in [15], demonstrating the relevance of our phantom study to characterize the relationship between TI and region volume. In addition, we found that most TI actually behave like entropy with respect to their dependency on the tumor volume, with high dependency on volume for small volume and then stable TI values beyond a certain volume. Still, the volume from which the plateau was reached varied from one TI to another (see Fig 3A and 3C and S1A, S1C and S1G Fig). We also observed that some TI never reached a plateau, confirming that these indices were poor descriptors of texture (RLNU, see [14]).

We also found that the relationship between TI and MV was similar in phantom data and in clinical data (Fig 3), demonstrating for the first time that the phantom approach was appropriate to characterize the TI dependency on volume as observed in patients, in spite of differences in SUV distribution (Fig 3) and signal-to-noise ratios between the phantom and clinical data (phantom: 11 kBq/mL at scan time scanned for 3 min; patient: 3–3.5 kBq/mL at scan time scanned for 1.45 min per bed position).

In their conclusion, Brooks and Grigsby [15] recommended calculating entropy only in regions greater than 45 cm^3^, which significantly limits the use of TI. The alternative AR resampling scheme we propose actually overcome this limitation and enable calculation of TI without introducing a large bias associated with the tumor volume. The rationale for the AR method is that the range of SUV in a given tumor actually already provides information regarding the tumor heterogeneity, regardless of the spatial arrangement of these values within a tumor. Indeed, it is expected that if a tumor region includes a broad range of SUV values, the tumor will be more heterogeneous than if all SUV are similar. Therefore, removing the SUV range information by using Eq (1) in the resampling step does not appear to be relevant for assessing the tumor texture. On the contrary, the fact that TI are correlated with SUV (Fig 4) does not appear counterintuitive since the range of SUV in a tumor actually already reflects some tumor heterogeneity: for instance, if SUV varies from 2 to 10 in a tumor, it is expected to be more heterogeneous than a tumor in which SUV varies only from 2 to 3.

Resampling between fixed low and high SUV bounds therefore preserves this useful SUV information in the TI calculation. We arbitrarily chose 0 as a low bound and 20 as a high bound, as this is typically the range of SUV encountered in the clinics. To assess the impact of the arbitrary high bound value, we investigated the robustness of our results when changing the higher bound to 15 or 25 SUV units (Tables 2 and 4) and found that the conclusions remained unchanged, demonstrating that the choice of this high bound is not critical.

Using AR-based TI, it is possible to determine whether textural features are different as a function of the tissue type, without being affected by differences in the volume of the analyzed tissue. As a first *in vivo* model that could serve as a toy model, we first investigated whether TI were significantly different in healthy and tumor tissue. Although it is obvious that this distinction is not clinically meaningful, TI should enable the distinction between healthy and tumoral tissues if they are expected to be useful to distinguish between subtle differences in tissue types. Using this toy model, we demonstrated that all 7 TI were significantly different between these two tissue types when using the AR method, while this was not the case when using the RR method. Healthy tissue was more homogeneous than tumor tissue (Table 3) as shown by higher homogeneity and lower entropy values in the liver compared to the tumor in our NSCLC patients. Similarly, we observed that tumors contained less “low gray-level” and more “high gray-level” voxels than healthy tissue (Table 3), corresponding to lower LGZE and higher HGZE in tumors, which is again what could be intuitively expected. To the best of our knowledge, only two studies compared the range of TI across tissue types so far. Using 103 untreated patients with bone and soft-tissue lesions, Xu et al [23] showed that TI from PET and CT images could differentiate between malignant and benign lesions. They found that entropy from FDG-PET images was higher in malignant tumors than in benign lesions. The second study [24] reported that the contrast TI (from CM) was greater in the liver compared to tumors for 11 patients with breast cancer who underwent a fluorothymidine (FLT) PET scan before treatment. In our study, we found that the same contrast index (data not shown) was higher in lung tumors (median 26.0, range: [1.2–219.7]) than in liver tissue (median 1.1, range: [0.5–2.8]). These discrepant results might be explained by the use of different tracers, by the low number of patients in [24], by the inclusion of tumors with very heterogeneous volumes (2.65 to 540.99 mL) without accounting for these differences in volumes and by the use of RR instead of AR. It is important to underline that when using RR, none of the TI yielded a pvalue lower than that of SUVmax, while when using the AR method, 4 out of the 7 TI did better than SUVmax at distinguishing between tumor and healthy tissue, demonstrating the relevance of the AR method. Given that AR-based TI also reflect some SUV information, we checked how the pvalues of Wicoxon’s tests of AR-based TI compared with the pvalues of the combination between RR-based TI and SUVmax (S3 Table). We found that the AR-based TI led to very similar results compared to RR-based TI associated with SUVmax using a logistic regression with lower pvalues for homogeneity, entropy, SRE and LGZE to differentiate tumor and healthy tissue. An advantage of using AR-based TI is that it consistently includes TI and SUV information into a single index, which might facilitate its use in a clinical setting compared to the co-analysis of two indices (RR-based TI and SUV).

When determining whether TI were different between tumor subtypes, we again observed that with the AR method, tumor subtypes were better distinguished than with the RR method (Table 4). TI reflected differences between FDG uptake pattern in adenocarcinomas and squamous cell carcinomas: the adenocarcinomas were more homogeneous, with higher homogeneity and lower entropy, and had less “high gray-level” voxels than the squamous cell carcinomas (Table 3). Chi-2 Pearson tests showed that only the presence of necrosis measured on the resected specimen was significantly different between the two subtypes of cancer (pvalue<0.01), unlike T stage, N stage and the level of differentiation (Table 5). We can thus hypothesize that AR-based TI could capture the presence of histologic necrosis. In their study, Asamura et al [25] demonstrated that patients with squamous cell carcinomas had a poorer prognosis than those with adenocarcinomas, after surgical resection, based on 11,939 patients. Moreover the squamous cell carcinomas exhibit a high proliferative index compared to the adenocarcinomas [26]. Altogether, these results suggest than AR-based TI might assist in objectively assessing tumor aggressiveness by efficiently combining texture information and SUV in a single index. For three TI (homogeneity, entropy and LGZE), the use of AR-based TI led to a better distinction between the tumor types than combining RR-based indices with SUVmax (S3 Table).

**Table 5:** Tumor characteristics from tumor specimen after surgery and pvalues of Chi-2 Pearson test between two subtypes.

Characteristic (pvalue of Chi-2 Pearson test)		Adenocarcinomas	Squamous cell carcinomas
T stage (0.5816)	T1	8	3
	T2	15	7
	T3	3	3
	T4	2	0
N stage (0.8856)	N0	8	9
	N+	20	4
Differentiation (0.9438)	Well	15	7
	Moderately or poorly	4	2
	N/A	9	4
Presence of necrosis (**0.0018**)	Yes	13	0
	No	13	13
	N/A	2	0

N/A: not available.

Pvalues in bold are lower than 5%.

The AR method is close to the resampling method used by Leijenaar et al [19], where the authors resampled voxel intensities with fixed bin width of 0.5 SUV units. Their approach actually corresponds to using AR with the maximum bound of 32 SUV units. With a maximum bound of 20 SUV units and 64 discrete values, the bin-width is approximately of 0.3 SUV units, which makes the TI sensitive to smaller variations in voxel SUV compared to a bin width of 0.5 SUV units. The optimal bin size could be further investigated, but we already demonstrated that our results did not strongly depend on it (Tables 2 and 4).

Our results should be confirmed using larger cohorts, since we only studied the distinction of subtypes between 28 adenocarcinomas and 13 squamous cell carcinomas. Moreover, our conclusions should be validated using different cancer types, and a next step will be to establish expected normal TI values as a function of the tissue type, so that TI could be used prospectively on an individual patient basis to assess the tumor homogeneity, instead of being only used in retrospective studies as has been reported so far.

On-going standardization of PET imaging protocols should make it easier to later define some common rules for TI interpretation, also accounting for TI variability as a function of the PET acquisition mode and reconstruction parameters previously described by Galavis et al [13]. Moreover, as previously underlined [14], the relevance of TI should always be studied by measuring its incremental value for tumor characterization with respect to the widely used SUV and MV.

To conclude, textural patterns of metabolic activity can be measured without being biased by the region volume by using an absolute resampling method identical for all tumors and preserving the SUV information that already reflect some tumor heterogeneity. We demonstrated that AR-based TI were significantly different in lung tumors and healthy tissue and also in squamous cell carcinoma and adenocarcinoma, and that these differences were greater than those observed with SUVmax or MV. These results show that AR-based TI capture relevant information regarding the amplitude and spatial distribution of SUV and might be of interest to enrich the tumor profiling in radiomic analyses [27] that aim at a comprehensive characterization of tumors based on image features.

## Supporting Information

S1 Fig RR-based TI as a function of MV and SUVmax. Plots of SRE (A-B), LRE (C-D), RLNU (E-F) and LGZE (G-H) as a function of the number of voxels (A, C, E, G) or as a function of SUVmax (B, D, F, H) for the phantom (blue), lung tumors (red) and healthy tissue (green) with the relative resampling.
(TIF)

S2 Fig AR-based TI as a function of MV and SUVmax. Plots of SRE (A-B), LRE (C-D), RLNU (E-F) and LGZE (G-H) as a function of the number of voxels (A, C, E, G) or as a function of SUVmax (B, D, F, H) for the phantom (blue), lung tumors (red) and healthy tissue (green) with the absolute resampling.
(TIF)

S1 Table Mean, first and third quartile (Q1, Q3) of RR-based and AR-based TI for tumor tissue and healthy tissue. (TIF)

S2 Table Mean, first and third quartile (Q1, Q3) of RR-based and AR-based TI for adenocarcinoma and squamous cell carcinoma. (TIF)

S3 Table Pvalues of Wilcoxon’s test for the combination between RR-based texture indices and SUVmax for the differentiation between tumor and healthy tissue and between adenocarcinoma and squamous cell carcinoma. (TIF)
