# Supplementary figures and images for: 18F-FDG PET-Derived Textural Indices Reflect Tissue-Specific Uptake Pattern in Non-Small Cell Lung Cancer
Source: PLoS One. 2015 Dec 15;10(12):e0145063. doi: 10.1371/journal.pone.0145063 (PMC4682929; doi:10.1371/journal.pone.0145063)

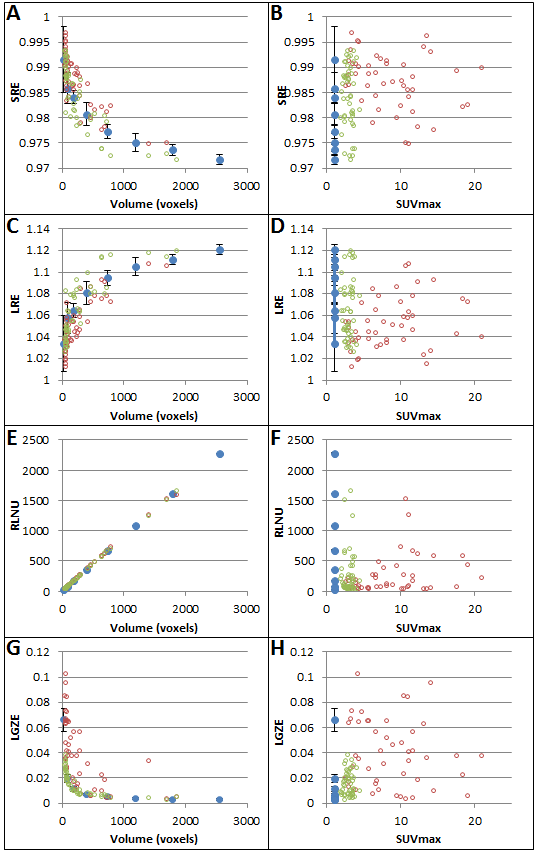

Supplement: S1 Fig — Plots of SRE (A-B), LRE (C-D), RLNU (E-F) and LGZE (G-H) as a function of the number of voxels (A, C, E, G) or as a function of SUVmax (B, D, F, H) for the phantom (blue), lung tumors (red) and healthy tissue (green) with the relative resampling. (TIF) [file pone.0145063.s001.tif]

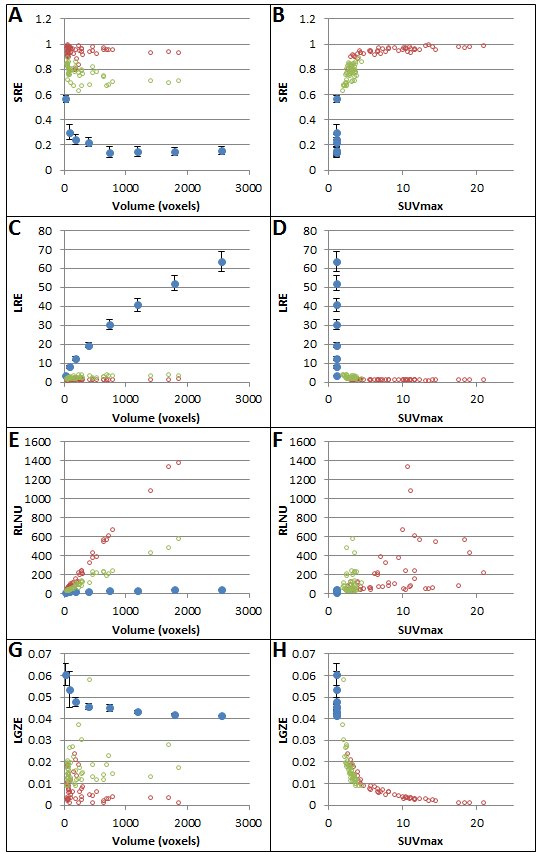

Supplement: S2 Fig — Plots of SRE (A-B), LRE (C-D), RLNU (E-F) and LGZE (G-H) as a function of the number of voxels (A, C, E, G) or as a function of SUVmax (B, D, F, H) for the phantom (blue), lung tumors (red) and healthy tissue (green) with the absolute resampling. (TIF) [file pone.0145063.s002.tif]

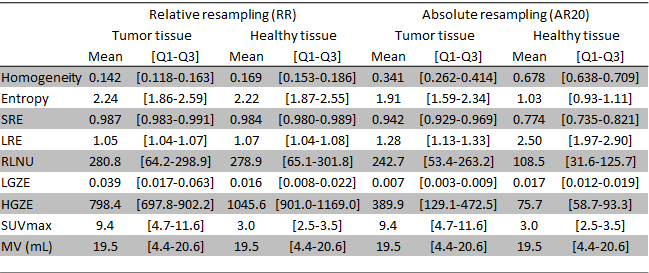

Supplement: S1 Table — (TIF) [file pone.0145063.s003.tif]

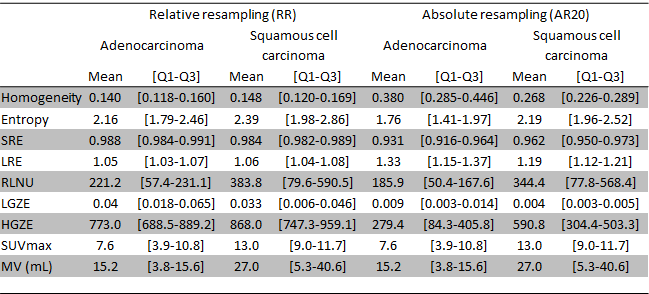

Supplement: S2 Table — (TIF) [file pone.0145063.s004.tif]

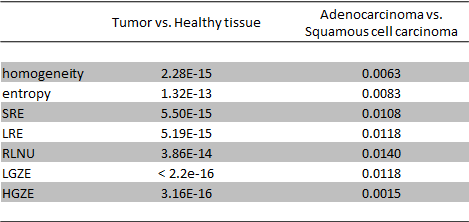

Supplement: S3 Table — (TIF) [file pone.0145063.s005.tif]
